# In Vitro Susceptibility to Ceftazidime-Avibactam and Comparator Antimicrobial Agents of Carbapenem-Resistant Enterobacterales Isolates

**DOI:** 10.3390/microorganisms11092158

**Published:** 2023-08-25

**Authors:** Tze-Peng Lim, Jun-Yuan Ho, Jocelyn Qi-Min Teo, James Heng-Chiak Sim, Si-Hui Tan, Thuan-Tong Tan, Andrea Lay-Hoon Kwa

**Affiliations:** 1 Department of Pharmacy, Singapore General Hospital, 10 Hospital Boulevard, Singapore 168582, Singapore; 2 SingHealth Duke-NUS Pathology Academic Clinical Programme, 20 College Road, Singapore 169856, Singapore; james.sim@parkwaylabs.com.sg; 3 SingHealth Duke-NUS Medicine Academic Clinical Programme, 10 Hospital Boulevard, Singapore 168582, Singapore; 4 Department of Microbiology, Singapore General Hospital, 20 College Road, Singapore 169856, Singapore; 5 Department of Infectious Diseases, Singapore General Hospital, 20 College Road, Singapore 169856, Singapore; 6 Emerging Infectious Diseases Program, Duke-NUS Medical School, 8 College Road, Singapore 169857, Singapore

**Keywords:** ceftazidime/avibactam, antibiotic susceptibility, CRE, minimum inhibitory concentrations, carbapenemase

## Abstract

The emergence of carbapenem-resistant Enterobacterales (CRE) has been recognized as a significant concern globally. Ceftazidime/avibactam (CZA) is a novel β-lactam/β-lactamase inhibitor that has demonstrated activity against isolates producing class A, C, and D β-lactamases. Here-in, we evaluated the in vitro activity of CZA and comparator antimicrobial agents against 858 CRE isolates, arising from the Southeast Asian region, collected from a large tertiary hospital in Singapore. These CRE isolates mainly comprised *Klebsiella pneumoniae* (50.5%), *Escherichia coli* (29.4%), and *Enterobacter cloacae* complex (17.1%). Susceptibility rates to levofloxacin, imipenem, meropenem, doripenem, aztreonam, piperacillin/tazobactam, cefepime, tigecycline, and polymyxin B were low. CZA was the most active β-lactam agent against 68.9% of the studied isolates, while amikacin was the most active agent among all comparator antibiotics (80% susceptibility). More than half of the studied isolates (51.4%) identified were *Klebsiella pneumoniae* carbapenemase (KPC)-2 producers, 25.9% were New Delhi metallo-β-lactamase (NDM) producers, and Oxacillinase (OXA)-48-like producers made up 10.7%. CZA was the most active β-lactam agent against KPC-2, OXA-48-like, and Imipenemase (IMI) producers (99.3% susceptible; MIC_50/90_: ≤1/2 mg/L). CZA had excellent activity against the non-carbapenemase-producing CRE (91.4% susceptible; MIC_50/90_: ≤1/8 mg/L). Expectedly, CZA had no activity against the metallo-β-lactamases (MBL)-producing CRE (NDM- and Imipenemase MBL (IMP) producers; 27.2% isolates), and the carbapenemase co-producing CRE (NDM + KPC, NDM + OXA-48-like, NDM + IMP; 3.0% isolates). CZA is a promising addition to our limited armamentarium against CRE infections, given the reasonably high susceptibility rates against these CRE isolates. Careful stewardship and rational dosing regimens are required to preserve CZA’s utility against CRE infections.

## 1. Introduction

Carbapenem-resistant Enterobacterales (CRE) infections are associated with prolonged hospitalizations, higher healthcare costs, and increased mortality rates [1,2,3,4,5]. Polymyxin B/colistin and tigecycline are considered the last-resort antibiotics against CRE infections. However, they are associated with poor pharmacokinetic/pharmacodynamic (PK/PD) characteristics and therefore compromise the safety and efficacy of antibiotic treatment [6]. Moreover, these CRE isolates also present resistance towards other classes of antibiotics such as fluoroquinolones, aminoglycosides, and tetracyclines [7]. Therefore, infections caused by CRE are a significant public health issue in Singapore, and worldwide. According to a point prevalence survey on healthcare-associated infections in 2015, approximately 7% of all nosocomial infections caused by Enterobacterales in Singapore were carbapenem-resistant [8]. Therefore, the most crucial strategy against CRE will be preventing its spread in the long term. Until then, patients will continue to present with difficult-to-treat infections caused by CRE.

A variety of mechanisms mediate carbapenem resistance in Enterobacterales—these include the synthesis of carbapenemase enzymes, extended-spectrum β-lactamases (ESBLs), AmpC enzymes (mainly plasmid-mediated), over-expressed efflux pumps (extrudes antibiotics) and porin loss [7]. However, the most frequently reported carbapenem resistance mechanism is the production of carbapenemases among CRE. Carbapenemases belong to three molecular classes of β-lactamases, Ambler class A, B, and D [9,10]. Using the Ambler classification system, carbapenemases are found within class A, B, and D β-lactamases, with substantial diversity in classes across geographic regions [10,11,12]. Therefore, exploring new and viable treatment options for these life-threatening infections is essential. 

Ceftazidime/avibactam (CZA) is a novel β-lactam/β-lactamase inhibitor combination approved by the U.S. Food and Drug Administration and became available for use in Singapore in 2018. It has in vitro activity against Ambler class A β-lactamases, e.g., *Klebsiella pneumoniae* carbapenemases (KPCs), Ambler class C β-lactamases (e.g., AmpC), and some Ambler class D β-lactamases (e.g., Oxacillinase (OXA)-48-like), but not against class B metallo-β-lactamases (MBL) such as Imipenemase MBL (IMP), Verona Integron-encoded MBL (VIM), and New Delhi MBL (NDM) [13]. In addition, several in vitro and clinical studies have supported CZA therapy against CRE infections [14,15]. Singapore is an island nation with a diverse population of 5.6 million people [16]. The country is particularly susceptible to the risk of antimicrobial resistance (AMR), being an international travel and medical tourism hub in Southeast Asia. The lack of surveillance data from this region has limited the understanding of the clinical utility of CZA. Therefore, the objective of this study was to evaluate the in vitro activity of CZA and comparator antimicrobial agents available for use against CRE in this geographic region.

## 2. Materials and Methods

### 2.1. Bacterial Isolates

The majority of the CRE isolates from various clinical specimens were collected from a large tertiary hospital (>1800 beds) in Singapore between 2008 and 2021 as part of a hospital-wide surveillance study on carbapenem-resistant Gram-negative bacteria (initially obtained from Singapore General Hospital’s Diagnostic Bacteriology Laboratory). The remaining isolates were received at Singapore General Hospital Antimicrobial Resistance Research Laboratory for antibiotic combination testing [17]. These include isolates from patients in other local hospitals and foreign patients from regional countries around Singapore seeking medical treatment [18,19].

The genus identity was determined at the hospitals’ microbiology laboratory as part of routine investigations using VITEK^®^ GNI+ cards (bioMérieux, Hazelwood, MO, USA) or matrix-assisted laser desorption ionization time-of-flight mass spectrometry (MALDI-TOF MS) (Bruker Daltonik, Bremen, Germany). Carbapenem susceptibility was determined as part of routine investigations at the microbiology laboratory through the disc diffusion method, gradient diffusion method, or the VITEK^®^ 2 system (bioMérieux, Hazelwood, MO, USA).

The isolates were stored at −70 °C in Microbank^TM^ storage vials (ProLab Diagnostics Inc., Richmond Hill, ON, Canada). Fresh isolates were subcultured twice on 5% sheep’s blood Agar plates (Thermo Fisher Scientific Microbiology, Melaka, Malaysia) for 24 h at 35 °C before each experiment.

### 2.2. Antimicrobial Susceptibility Testing, Interpretation and Screening for Carbapenemases

The minimum inhibitory concentrations (MICs) to various antibiotics were determined using commercial dehydrated broth microdilution panels (Trek Diagnostics, East Grinstead, UK) and performed according to the manufacturer’s recommendations. In brief, turbidity-adjusted bacterial suspensions from fresh overnight cultures were added to cation-adjusted Mueller–Hinton broth (Ca-MHB, BBL, Sparks, MD, USA) to achieve an inoculum of 5 × 10^5^ colony forming units (CFUs)/mL. A measure of 100 µL of the bacterial suspension was added to each well and subsequently incubated for 16–20 h at 35 °C. The tested antibiotics included amikacin, aztreonam, cefepime, CZA, doripenem, ertapenem, imipenem, levofloxacin, meropenem, piperacillin/tazobactam, polymyxin B, and tigecycline. *E. coli* ATCC 25922 was used as the quality control strain. MICs were interpreted according to the Clinical and Laboratory Standards Institute (CLSI) M100 guidelines (33rd edition), except for tigecycline, which was interpreted according to the European Committee on Antimicrobial Susceptibility Testing (EUCAST, Version 13) criteria for tigecycline [20,21].

Carbapenemase genotypic characterization was performed using whole-genome sequencing as previously described [22]. In brief, genomic deoxyribonucleic acid (DNA) was extracted and purified from overnight bacterial cultures with the DNeasy blood and tissue kit (Qiagen GmbH, Hilden, Germany) according to the manufacturer’s protocol. Whole-genome sequencing was performed using the Illumina HiSeq or MiSeq system (Illumina, San Diego, CA, USA). Paired-end whole-genome sequencing (WGS) was performed on the genomic DNAs using the MiSeq/HiSeq systems (Illumina Inc., San Diego, CA, USA), with a resultant coverage of at least 100-fold. Raw sequences were assessed for quality using FastQC (v0.11.3, Babraham Institute), followed by the removal of adaptors and poor-quality bases using Trimmomatic [23,24]. Trimmed sequences were then assembled de novo using SPAdes software [25]. The antimicrobial resistance genes were identified using the AMRFinder tool [26].

## 3. Results

### 3.1. Bacteria Isolates and Carbapenemase Type

A total of 858 CRE isolates were included in the study. *Klebsiella pneumoniae* (*n* = 433, 50.5%), *Escherichia coli* (*n* = 252, 29.4%), and *Enterobacter cloacae* complex (*n* = 147, 17.1%) were the most common species. Rectal (*n* = 326, 38.0%), blood cultures (*n* = 138, 16.1%), urinary tract (*n* = 103, 12.0%), tissue (*n* = 100, 11.7%) intra-abdominal (*n* = 93, 10.8%), respiratory (*n* = 87, 10.1%), and bone (*n* = 11, 1.3%) were the specimen sources of the isolates.

**Figure 1:**
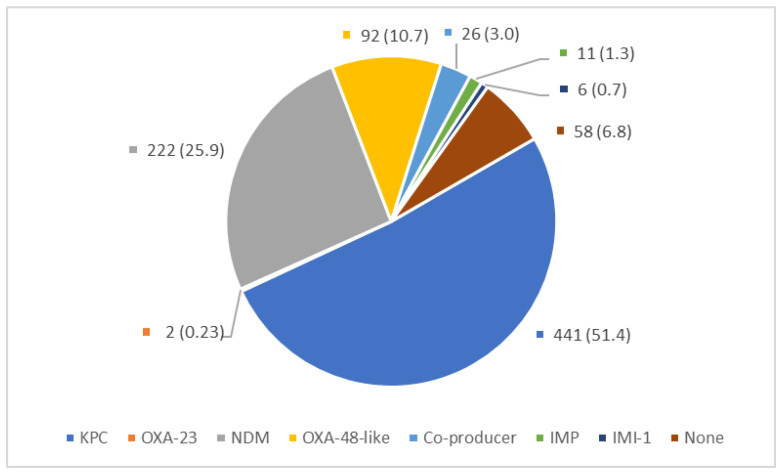
Carbapenemase types among the studied isolates (*n* = 858); *n* (%).

**Figure 2:**
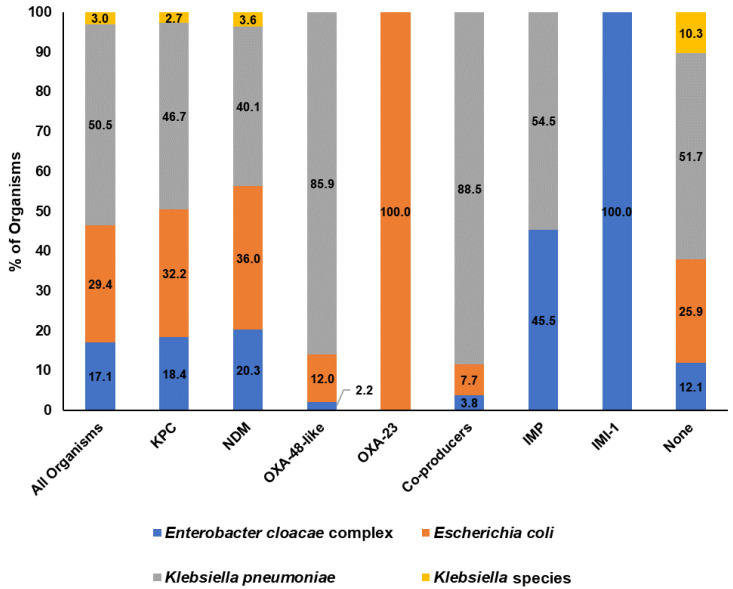
Bacteria species distribution by carbapenemase producer type.

The carbapenemase types of the studied isolates are shown in Figure 1. Carbapenemase genes were positive in 93.2% (800/858) of the CRE isolates. More than half of the isolates (51.4%, 441/858) were KPC producers, 25.9% (222/858) were NDM producers, and OXA-48-like producers made up 11.0%. The remaining isolates (11.8%) comprised IMP producers, carbapenemase co-producers (isolates that harbor more than one carbapenemase), IMI producers, and non-carbapenemase producers. The distribution of the bacterial species by carbapenemase types is shown in Figure 2. Compared to the overall species breakdown, we observed similar species composition trends—about half were *K. pneumoniae* in KPC producers, NDM producers, IMP producers, and non-producers. Interestingly, *K. pneumoniae* represented more than 80% of OXA-48-like producers (79/94) and co-producers (23/26). In addition, all IMI producers were *Enterobacter cloacae* complex, albeit only six isolates were included in this study. KPC-2 was the only KPC variant identified among KPC producers. NDM-1 was the predominant NDM-type carbapenemase, representing 77.0% (171/222) among all NDM producers. Other NDM variants include NDM-4 (1.8%, 4/222), NDM-5 (11.7%, 26/222), NDM-7 (5.0%, 11/222) and unknown NDM variants (4.5%, 10/222). Among the OXA-48-like producers, OXA-232 (44.6%, 41/92) was the predominant OXA-48-like variant, followed by OXA-181 (33.7%, 31/92), and OXA-48 (21.7%, 20/92). Interestingly, there were two *E. coli* isolates that harbored OXA-23 carbapenemases. The carbapenemase co-producing CRE mainly harbored NDM (NDM-1, NDM-5 or NDM-7) and OXA-48-like genes (OXA-48, OXA-181 or OXA-232) (88.5%, 23/26), while the remaining three isolates had KPC-2 and NDM-1 (two isolates) and IMP-1 and NDM-1 (one isolate).

### 3.2. Antimicrobial Susceptibility Testing and Interpretation

The CRE susceptibilities to the tested antibiotics are displayed in Table 1. A total of 68.9% (591/858) of the CRE isolates were susceptible to CZA. Amikacin, CZA, and tigecycline (79.8%, 68.9%, and 57.7% susceptible, respectively) were the most active antimicrobial agents against the CRE tested. CZA MICs ranged from ≤1 mg/L to ≥128 mg/L, and MIC_50_ and MIC_90_ were ≤1 mg/L and ≥128 mg/L, respectively. In addition, 25.2% (216/858) of the isolates retained susceptibility to levofloxacin, while only 6.8% (58/858) were susceptible to aztreonam. More than 95% of the isolates were non-susceptible to the rest of the tested antibiotics. Although there is no categorical interpretative susceptibility for polymyxin B, more than 16.3% (140/858) of the studied isolates exhibited high polymyxin B MICs ranging from 4 to ≥16 mg/L.

For all species of Enterobacterales, CZA has the highest susceptibility (64.6–72.1%) when compared with other β-lactam antibiotics (Table 2 and Supplementary Figure S1A–C)). However, amikacin susceptibility among all comparator antibiotics is generally the highest against most of the isolates. This was observed in *Enterobacter cloacae* complex (91.2%), *Klebsiella pneumoniae* (70.4%), *Escherichia coli* (88.9%), and *Klebsiella* species (84.6%). Tigecycline has the highest susceptibility rate against *Escherichia coli* at 92.1%.

CZA exhibited excellent activity against KPC-, OXA-48-like-, and IMI producers, with a combined susceptibility rate of 99.3% (Table 3). In addition, it was also highly active against carbapenemase non-producers and achieved 91.4% (53/58) susceptibility. Expectedly, CZA has no in vitro activity against MBL-producing CRE (NDM- and IMP producers). It is also inactive against the carbapenemase co-producing CRE in our study as they primarily co-produce NDM with another carbapenemase. Nine isolates that were CZA resistant either produced KPC-2 or OXA-232 carbapenemases or did not harbor any carbapenemases. These isolates also co-produce a variety of ESBLs and AmpC. These include ACT, SHV, EC, CMY, OXA, CTX-M, TEM, and DHA (Table 4).

## 4. Discussion

CRE infections have been recognized as a significant concern globally. Clinicians worldwide have already been confronted with the reality of infections caused by CRE, which are usually resistant to almost all current antibiotics. Our study and previous international reports have shown that CZA is not active against MBL-producing Enterobacterales, a known significant gap of avibactam’s activity spectrum [27,28,29]. However, it is very active against KPC-producing, OXA-48-like-producing, IMI-1-producing, and non-carbapenemase-producing CRE isolates, displaying >90% overall susceptibility. Therefore, carbapenemase detection at the clinical laboratory is crucial to tailor CZA treatment against CRE infections. This is exemplified by the diverse carbapenemase epidemiology observed in our CRE collection, with ~50% KPC producers and ~25% NDM producers. Most major clinical laboratories in Singapore provide carbapenemase detection as a clinical service. Therefore, CZA can be considered a front-line treatment option for treating these CRE infections in our setting.

Of note, we observed a small subset of our studied isolates (*n* = 39, comprising *K. pneumoniae* and *E. coli*) that showed reduced susceptibility to CZA (≥4 to 8 mg/L). They harbor either *bla*_KPC-2_ (18/39), *bla*_OXA-48-like_ genes (10/39) or no carbapenemase genes (11/32). A total of 32/39 were from bloodstream, respiratory, tissue, urine, and intra-abdominal infections, while the remaining isolates were from rectal sources. Although KPC-3 producers have grown prevalent worldwide and are the predominant variant in the United States, Italy, and Israel [30], the KPC producers in our study all harbor *bla*_KPC-2_. These 39 isolates also harbored a variety of ESBLs including CMY-2, CMY-42, CTX-M-15, CTX-M-55, CTX-M-65, DHA-1, EC, OXA-1, OXA-2, OXA-9, OXA-21, PER-7, SHV-1, SHV-11, SHV-12, SHV-28, SHV-62, TEM-1 and AmpC. They harbored a range of one to eight ESBLs for each isolate, and the median number of ESBLs was three. In addition, the nine CZA-resistant isolates (CZA MIC: 16 ≥ 128 mg/L) that did not harbor any MBLs also co-produced numerous ESBLs. The presence of CTX-M-15 or AmpC mutations and porin mutations in the *ompK35/36* gene in conjunction with KPC-2 or OXA-48-like carbapenemases, giving rise to CZA resistance, has been reported [31]. Hence, we speculated that the wide repertoire of the ESBLs with the presence or absence of non-MBL carbapenemases may have partially contributed to varying degrees of decreased CZA susceptibility observed in these isolates.

These findings suggested that on top of routine carbapenemase detection, the optimal use of CZA should be guided by routine MIC testing and therapeutic drug monitoring—this is necessary to ensure that appropriate CZA dosing is employed, especially in septic patients with fluctuating hemodynamics infected by a CRE with reduced CZA susceptibility [32]. While routine carbapenemase detection may help in the choice of CZA treatment when the antibiogram is not yet available, CZA resistance in KPC producers may be underestimated due to the detection limitations of KPC variants associated with CZA resistance [33]. Hence, rapid tests to assess CZA susceptibility, especially for more severe cases of infection (e.g., sepsis), can be implemented to achieve a rapid response before even receiving the definitive antibiogram. Such approaches include European Committee on Antimicrobial Susceptibility Testing rapid antimicrobial susceptibility testing (EUCAST-RAST) and direct-Etest (DET)-RAST [34]. EUCAST RAST is a rapid and accurate method that can rapidly determine the CZA susceptibility of *K. pneumoniae* and *E. coli* directly from blood culture bottles, while DET-RAST has the advantage of determining MIC values with MIC reading after 8 h of incubation. In addition, our study included a small subset of carbapenemase co-producing CRE isolates. A total of 21/26 isolates were from clinical sources. They were mainly *Klebsiella pneumoniae* (20/23) and *Enterobacter cloacae* complex (1/26). They harbored *bla*_NDM_ plus *bla*_KPC-2_ or *bla*_OXA-48-like_ genes and were resistant to all the β-lactams tested. CZA resistance is driven primarily by the presence of the *bla*_NDM_ gene. The combination of CZA plus aztreonam may be a promising therapeutic option against carbapenemase co-producing and NDM-producing CRE infections [35,36,37].

Polymyxins and tigecycline are often used as a first-line treatment for CRE infections, owing to their susceptibility profiles against CRE. Overall, our studied isolates possessed low polymyxin B and tigecycline MICs (83.6% of studied isolates have polymyxin B MIC of ≤2 mg/L; overall tigecycline MIC_90_: 2 mg/L), similar to internationally published data [38,39]. However, expert recommendations have opined the avoidance of polymyxin use due to increased nephrotoxicity [6]. In addition, tigecycline monotherapy is generally limited to treating intra-abdominal infections as there are limited urine concentrations and poor serum/lung concentrations are achieved [6]. While amikacin was the most active antibiotic tested (80% susceptible) in the study, the excess nephrotoxicity associated with aminoglycoside-based regimens relative to newer β-lactam-β-lactamase inhibitor agents reduces the treatment utility against CRE infections other than catheter-related CRE bloodstream infections [6].

This study is not without limitations. We did not examine the influence of CZA susceptibility caused by porin loss and efflux pump expression in this study. Our studied isolates were mainly obtained from a single institution through a hospital-wide surveillance study. They may not fully represent all isolates in Singapore or from the neighboring region. However, the carbapenemase distribution shown in this study is similar to our local CRE epidemiology [8]. This diverse epidemiology is likely the consequence of the importation and exportation of carbapenemase-producing Enterobacterales among Southeast Asian countries, as they house most of the world’s population and are major medical tourism destinations and venues for vacationers [40]. The results obtained from this study provide comprehensive information on the utility of CZA treatment locally and in the Southeast Asian region.

## 5. Conclusions

In conclusion, CRE isolates collected as part of our hospital-wide surveillance study showed the highest susceptibility rates to amikacin and CZA among the antimicrobials tested. In addition, CZA susceptibility was the highest amongst the β-lactams tested and the MBL-negative isolates. These findings suggest that CZA is a reasonable treatment option for managing CRE infections in our local and regional settings. Considering the considerable prevalence of MBL-producing CRE, CZA therapy should be considered alongside phenotypic/genotypic testing to exclude the presence of intrinsically resistant CZA CRE infections in the respective clinical settings.

## Figures and Tables

**Table 1:** Susceptibility of CRE (*n* = 858) to CZA and comparator antibiotics.

Antibiotics	No. of Isolates (%)			MIC_50_ (mg/L)	MIC_90_ (mg/L)
	S	I	R		
Amikacin	685 (79.8)	19 (2.2)	154 (17.9)	≤4	≥128
Aztreonam	58 (6.8)	18 (2.1)	782 (91.1)	≥64	≥64
Cefepime	24 (2.8)	71 (8.3)	763 (88.9)	≥64	≥64
CZA	591 (68.9)	-	267 (31.1)	≤1	≥128
Doripenem	40 (4.7)	101 (11.8)	717 (83.6)	8	≥32
Ertapenem	0	11 (1.3)	847 (98.7)	≥32	≥32
Imipenem	9 (1.0)	31 (3.6)	818 (95.4)	16	≥32
Levofloxacin	216 (25.2)	128 (14.9)	514 (59.9)	8	≥64
Meropenem	24 (2.8)	38 (4.4)	796 (92.8)	16	≥32
Piperacillin/Tazobactam	9 (1.0)	14 (1.6)	835 (97.3)	≥128	≥128
Polymyxin B	-	718 (83.7)	140 (16.3)	0.5	≥16
Tigecycline	495 (57.7)	-	363 (42.3)	0.5	2

S: susceptible; I: intermediate; R: resistant; MIC: minimum inhibitory concentration; CZA: ceftazidime/avibactam.

**Table 2:** Susceptibility of CRE (*n* = 858) by species to CZA according to minimum inhibitory concentration (MIC) (mg/L) distribution and susceptibility to comparator antibiotics.

Bacteria Species	No. of Isolates (*n* = 858)	CZA									Susceptibility to Comparators (% Isolates Susceptible)										
		Cumulative Percentage of Isolates at Each MIC (mg/L)																			
		≤1	2	4	8	16	32	64	≥128	%S	AMK	AZT	FEP	DOR	ETR	IMI	LEV	MER	PT/4	PMB *	TGC
*Enterobacter cloacae* complex	147	53.1	63.9	64.6	64.6	65.3	65.3	65.3	100	64.6	91.2	8.2	4.8	4.1	0.0	0.0	29.9	2.7	3.4	0.0	63.9
*Escherichia coli*	252	58.3	61.1	63.1	66.3	66.3	66.3	66.3	100	66.3	88.9	10.3	2.4	10.3	0.0	1.2	27.4	5.2	0.8	0.0	92.1
*Klebsiella pneumoniae*	433	54.0	66.5	70.9	72.1	72.3	72.3	72.3	100	72.1	70.4	3.5	2.3	1.2	0.0	1.6	20.3	1.6	0.2	0.0	34.9
*Klebsiella* species	26	42.3	61.5	65.4	65.4	65.4	65.4	65.4	100	65.4	84.6	19.2	3.8	11.5	0.0	0.0	57.7	0.0	3.8	0.0	69.2

*: There is no susceptibility category defined for polymyxin B according to CLSI. AMK: Amikacin; AZT: Aztreonam; FEP: Cefepime; CZA: Ceftazidime/avibactam; DOR: Doripenem; ETR: Ertapenem; IMI: Imipenem; LEV: Levofloxacin; MER: Meropenem; PT/4: Pipercillin-Tazobactam; PMB: Polymyxin B; TGC: Tigecycline.

**Table 3:** Susceptibility of CRE (*n* = 858) by carbapenemase producers to CZA and comparator β-lactam antibiotics.

No. of Isolates (*n*)	Carbapenemase Type	% (*n*) Isolates Susceptible to Each Antibiotic							
		CZA	Ertapenem	Imipenem	Meropenem	Doripenem	Aztreonam	Piperacillin-Tazobactam	Cefepime
26	Co-producer *	0 (0)	0	0	0	0	7.7(2)	0	0
6	IMI ^a^	100 (6)	0	0	0	0	83.3 (5)	83.3 (5)	100 (6)
11	IMP	0 (0)	0	0	0	0	9.1 (1)	9.1 (1)	0
441	KPC ^b^	99.3 (438)	0	0.5 (2)	2.3 (10)	4.5 (20)	0.7 (3)	0.2 (1)	2.0 (9)
222	NDM ^c^	0.5 (1)	0	0	0	0	19.4 (43)	0	0
58	None	91.4 (53)	0	10.3 (6)	8.6 (5)	17.2 (10)	1.7 (1)	3.4 (2)	6.9 (4)
2	OXA-23	100.0 (2)	0	0	100.0 (2)	100.0 (2)	100.0 (2)	0	50.0 (1)
92	OXA-48-like ^d^	98.9 (93)	0	1.1 (1)	7.6 (7)	8.7 (8)	1.1 (1)	0	4.3 (4)

*: Isolates that harbor more than one carbapenemase. ^a^ IMI: variants identified include IMI-1 (*n* = 5) and new variants (*n* = 1) not included in the AMRFinder database [26]. ^b^ KPC: variants identified were all KPC-2 (*n* = 441) [26]. ^c^ NDM: variants identified include NDM-1 (*n* = 171), NDM-4 (*n* = 4), NDM-5 (*n* = 26), NDM-7 (*n* = 11) and new variants (*n* = 10) not included in the AMRFinder database [26]. ^d^ OXA-48-like: variants identified include OXA-48 (*n* = 20), OXA-181 (*n* = 31) and OXA-232 (*n* = 41) not included in the AMRFinder database [26].

**Table 4:** Characteristics of non-MBL-producing isolates (*n* = 9) that are resistant to CZA.

Organism	Carbapenemases	β-Lactamases	CZA MIC (mg/L)
*Enterobacter cloacae* complex	KPC-2	ACT-1, SHV-12	16
*Escherichia coli*	KPC-2	EC	≥128
*Escherichia coli*	None	CMY-42, EC, OXA-1	≥128
*Escherichia coli*	None	CTX-M-55, EC, TEM-1	≥128
*Klebsiella pneumoniae*	None	CTX-M-15, SHV-11, TEM-1, OXA-1	≥128
*Klebsiella pneumoniae*	OXA-232	CTX-M-15, TEM-1, OXA-1	≥128
*Klebsiella pneumoniae*	None	CTX-M-55, SHV-27, OXA-1	16
*Klebsiella pneumoniae*	KPC-2	CTX-M-65, LAP-2, SHV-12, TEM-1	≥128
*Klebsiella* species	None	AmpC, DHA-1	≥128

## Data Availability

The sequences from this study have been deposited in the NCBI Sequence Read Archive (SRA) BioProject under accession no. PRJNA577535.

## Supplementary Materials

The following supporting information can be downloaded at: https://www.mdpi.com/article/10.3390/microorganisms11092158/s1, Figure S1. CZA MIC distribution by organism. (**A**) CZA MIC Distribution of *Enterobacter cloacae* complex (n = 147); (**B**) CZA MIC Distribution of *Escherichia coli* (n = 252); (**C**) CZA MIC Distribution of *Klebsiella pneumoniae* (n = 433).
