# Supplementary material for: In Vitro Susceptibility to Ceftazidime-Avibactam and Comparator Antimicrobial Agents of Carbapenem-Resistant Enterobacterales Isolates
Source: Microorganisms. 2023 Aug 25;11(9):2158. doi: 10.3390/microorganisms11092158 (PMC10534512; doi:10.3390/microorganisms11092158)
Supplement: Supplementary file 1 [file microorganisms-11-02158-s001.zip › microorganisms-2475414-supplementary.pdf]

Supplementary Materials

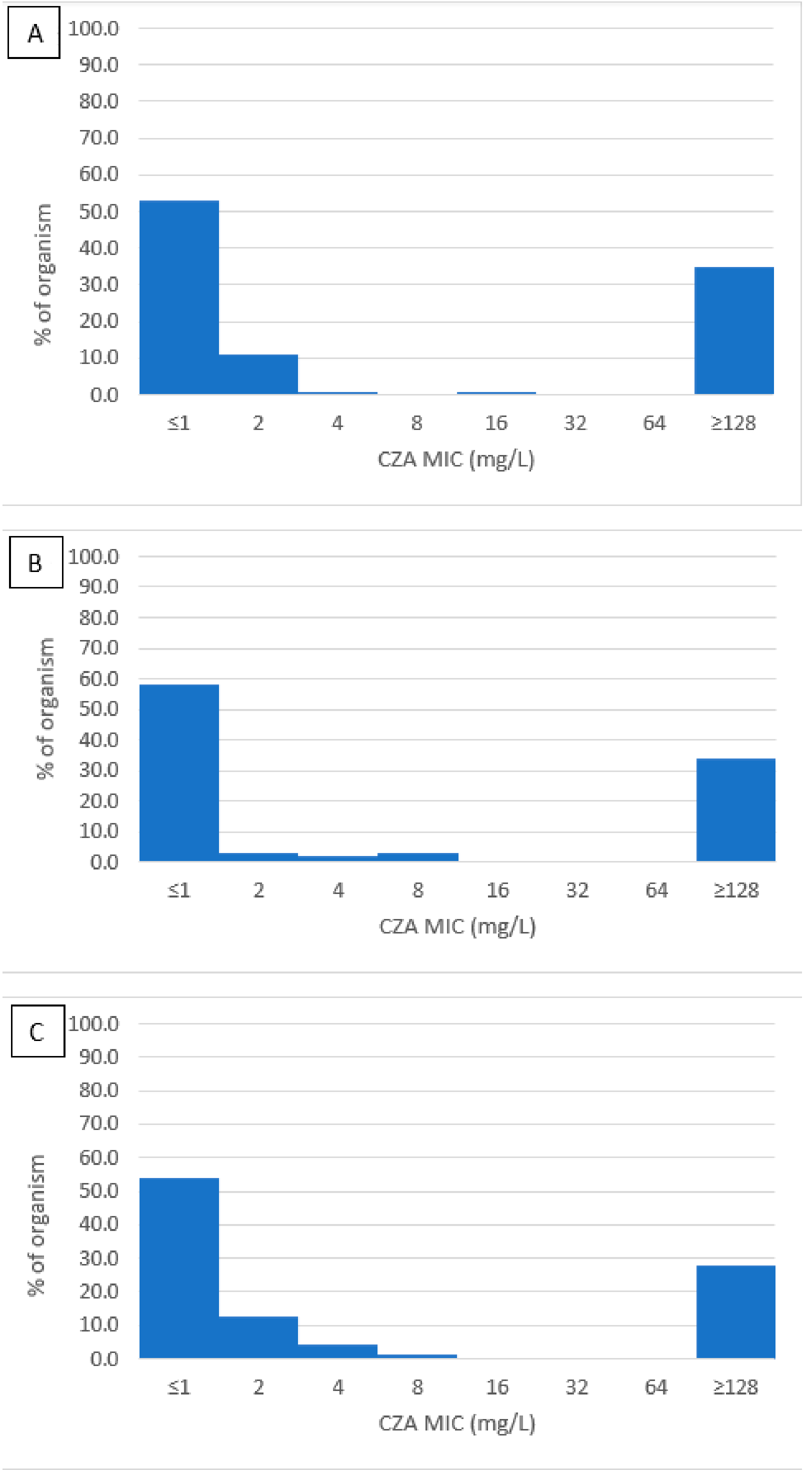

Figure S1. CZA MIC distribution by organism. (**A**) CZA MIC Distribution of *Enterobacter cloacae* complex (*n* = 147); (**B**) CZA MIC Distribution of *Escherichia coli* (*n* = 252); (**C**) CZA MIC Distribution of *Klebsiella pneumoniae* (*n* = 433).
